# Supplementary material for: Estimating error rates for single molecule protein sequencing experiments
Source: PLoS Comput Biol. 2024 Jul 5;20(7):e1012258. doi: 10.1371/journal.pcbi.1012258 (PMC11253918; doi:10.1371/journal.pcbi.1012258)
Supplement: S1 Appendix — (PDF) [file pcbi.1012258.s001.pdf]

S1 Appendix: Technical details of Baum-Welch

The Baum-Welch algorithm [17] is a form of Expectation Maximization [21], which will iterate over solutions to reach a maximum likelihood estimate. While only convergence to a local maximum likelihood can be guaranteed, it has been found that for most applications Expectation Maximization is nevertheless highly effective. Like all Expectation Maximization techniques, in Baum-Welch, we assume a parameterization to start, which we use to compute probabilities of intermediate hidden values, which we then use to produce a new and better estimate of the parameters. For Baum-Welch, this means iteratively improving the transition and emission probabilities.

The Baum-Welch algorithm is built partly upon the forward-backward algorithm. For any HMM we have a transition matrix and an emission matrix which define the model. Let $X_{1:T}$ represent the random variables which take one of N possible values over T time steps. Let $Y_{1:T}$ represent the random variables representing the emission distribution. Let s denote the step in our Baum-Welch iteration process, and $\theta^{(s)}$ represent our choice of parameters for step s, which includes our transition probabilities, emission distributions, and our initial conditions. Our transition matrix is given as:

$$T_{ji}^{(s)} = p\left(X_t = j \middle| X_{t-1} = i, \theta^{(s)}\right) \tag{4}$$

In most introductory descriptions of Baum-Welch, the emission values are from a discrete set, and an emission matrix is given as the probability of seeing a particular indexed output given a particular state. In this application, our emissions are floating point values, and we find it more clarifying to instead have a different emission matrix for every time step, and to think of it as a diagonal matrix where the values represent the probability

density of the known observation at that time step. Letting t denote the time step, and y_t denote the true observed value at time t. We define our emission matrix $O^{(s,t)}$ as follows:

$$O_{ii}^{(s,t)} = p\left(Y_t = y_t \middle| X_t = i, \theta^{(s)}\right) \tag{5}$$

We need to run the forward algorithm. We define our cumulative forward probabilities as:

$$f_i^{(s,t)} = p\left(Y_{1:t} = y_{1:t}, X_t = i \middle| \theta^{(s)}\right) \tag{6}$$

We can use dynamic programming to determine $f_i^{(s,t)}$ for every value of i and t, if given initial conditions $f_i^{(s,0)}$ for all i. The equation is:

$$f^{(s,t)} = O^{(s,t)} T^{(s)} f^{(s,t-1)} \tag{7}$$

Or equivalently:

$$p\left(Y_{1:t} = y_{1:t}, X_t = i \middle| \theta^{(s)}\right) = p\left(Y_t = y_t \middle| X_t = i, \theta^{(s)}\right) \sum_j p\left(X_t = i \middle| X_{t-1} = j, \theta^{(s)}\right) p\left(Y_{1:t-1} = y_{1:t-1}, X_{t-1} = j \middle| \theta^{(s)}\right) \tag{8}$$

We also need to run the backward algorithm. We define the probabilities as:

$$b_i^{(s,t)} = p\left(Y_{t+1:T} = y_{t+1:T} \middle| X_t = i, \theta^{(s)}\right) \tag{9}$$

We can use dynamic programming to determine $b_i^{(s,t)}$ for every value of i and t. We initialize it with:

$$b_i^{(s,T)} = 1 \tag{10}$$

And then compute:

$$b^{(s,t)} = {T^{(s)}}^{\top} O^{(s,t+1)} b^{(s,t+1)} \tag{11}$$

Or equivalently:

$$p\left(Y_{t+1:T} = y_{t+1:T} \middle| X_t = j, \theta^{(s)}\right) = \sum_i p\left(X_t = i \middle| X_{t-1} = j, \theta^{(s)}\right) p\left(Y_{t+1} = y_{t+1} \middle| X_{t+1} = i, \theta^{(s)}\right) p\left(Y_{t+2:T} = y_{t+2:T} \middle| X_{t+1} = i, \theta^{(s)}\right)$$

$$(12)$$

This gives us the capability to compute two sets of key intermediate values.

The first is the same as what is computed in the forward-backward algorithm and represents the probability of being in a state at a given time given the entire sequence of observations. It's defined as:

$$\gamma_i^{(s,t)} = p\left(X_t = i \middle| Y_{1:T} = y_{1:T}, \theta^{(s)}\right) \quad (13)$$

And computed with the formula:

$$\gamma_i^{(s,t)} = \frac{f_i^{(s,t)} b_i^{(s,t)}}{{f^{(s,t)}}^{\top} b^{(s,t)}} \quad (14)$$

Or equivalently:

$$p\left(X_t = i \middle| Y_{1:T} = y_{1:T}, \theta^{(s)}\right)$$
$$= \frac{p\left(Y_{1:t} = y_{1:t}, X_t = i \middle| \theta^{(s)}\right) p\left(Y_{t+1:T} = y_{t+1:T} \middle| X_t = i, \theta^{(s)}\right)}{\sum_j p\left(Y_{1:t} = y_{1:t}, X_t = j \middle| \theta^{(s)}\right) p\left(Y_{t+1:T} = y_{t+1:T} \middle| X_t = j, \theta^{(s)}\right)} \quad (15)$$

The next set of values represents the probability of a particular transition taking place between time step t and time step $t + 1$. We define it as:

$$\xi_{ij}^{(s,t)} = p\left(X_t = i, X_{t+1} = j \middle| Y_{1:T} = y_{1:T}, \theta^{(s)}\right) \quad (16)$$

We can compute this with:

$$\xi_{ij}^{(s,t)} = \frac{f_i^{(s,t)} T_{ij}^{(s)} O_{jj}^{(s,t+1)} b_j^{(s,t+1)}}{{f^{(s,t)}}^{\top} T^{(s)} O^{(s,t+1)} b^{(s,t+1)}} \quad (17)$$

Or equivalently:

$$p\left(X_t = i, X_{t+1} = j \middle| Y_{1:T} = y_{1:T}, \theta^{(s)}\right)$$
$$= \frac{p\left(Y_{1:t} = y_{1:t}, X_t = i \middle| \theta^{(s)}\right) p\left(X_{t+1} = j \middle| X_t = i, \theta^{(s)}\right) p\left(Y_{t+1} = y_{t+1} \middle| X_{t+1} = i, \theta^{(s)}\right) p\left(Y_{t+2:T} = y_{t+2:T} \middle| X_t = \text{[illegible]}\right.}{\sum_{\hat{\imath},\hat{\jmath}} p\left(Y_{1:t} = y_{1:t}, X_t = \hat{\imath} \middle| \theta^{(s)}\right) p\left(X_{t+1} = \hat{\jmath} \middle| X_t = \hat{\imath}, \theta^{(s)}\right) p\left(Y_{t+1} = y_{t+1} \middle| X_{t+1} = \hat{\imath}, \theta^{(s)}\right) p\left(Y_{t+2:T} = y_{t+2:T} \middle| X_t \text{[illegible]}\right.}$$

$$(18)$$

We then update our transition probabilities according to:

$$T_{ij}^{(s+1)} = \frac{\sum_t \xi_{ij}^{(s,t)}}{\sum_t \gamma_i^{(s,t)}} \tag{19}$$

Or the equivalent expression:

$$p\left(X_t = j \middle| X_{t-1} = i, \theta^{(s+1)}\right) = \frac{\sum_t p\left(X_t = i, X_{t+1} = j \middle| Y_{1:T} = y_{1:T}, \theta^{(s)}\right)}{\sum_t p\left(X_t = i \middle| Y_{1:T} = y_{1:T}, \theta^{(s)}\right)} \tag{20}$$

We also need to determine our new initial conditions. We solve for these as:

$$f_i^{(s+1,0)} = \gamma_i^{(s,0)} \tag{21}$$

To update our emission probabilities, we perform a weighted maximum likelihood estimate, using it to determine the appropriate weights. The form of the maximum likelihood estimate depends on the assumed shape of the output distribution, but if we assume each output distribution is normally distributed, where $Y_t \sim N\left(\mu_i^{(s)}, \sigma_i^{(s)}\right)$ when $X_t = i$, we can compute $\mu^{(s+1)}$ and $\sigma^{(s+1)}$ as:

$$\mu_i^{(s+1)} = \frac{\sum_t \gamma_i^{(s,t)} Y_t}{\sum_t \gamma_i^{(s,t)}} \tag{22}$$

$$\sigma_i^{(s+1)} = \sqrt{\frac{\sum_t \gamma_i^{(s,t)} \left(Y_t - \mu_i^{(s+1)}\right)^2}{\sum_t \gamma_i^{(s,t)}}} \tag{23}$$

Or the equivalent expressions:

$$\mu_i^{(s+1)} = \frac{\sum_t p\left(X_t = i \middle| Y_{1:T} = y_{1:T}, \theta^{(s)}\right) Y_t}{\sum_t p\left(X_t = i \middle| Y_{1:T} = y_{1:T}, \theta^{(s)}\right)} \tag{24}$$

$$\sigma_i^{(s+1)} = \sqrt{\frac{\sum_t p\left(X_t = i \middle| Y_{1:T} = y_{1:T}, \theta^{(s)}\right) \left(Y_t - \mu_i^{(s+1)}\right)^2}{\sum_t p\left(X_t = i \middle| Y_{1:T} = y_{1:T}, \theta^{(s)}\right)}} \tag{25}$$

We can then compute a new emission matrix using these new parameter estimates:

$$O_{ii}^{(s+1,t)} = N\left(y_t; \mu_i^{(s+1)}, \sigma_i^{(s+1)}\right) \tag{26}$$

This can be trivially generalized to multiple sequences. Let r denote the index of a sequence, ranging from 1 to R. Then:

$$T_{ij}^{(s+1)} = \frac{\sum_r \sum_t \xi_{ij}^{(s,r,t)}}{\sum_r \sum_t \gamma_i^{(s,r,t)}} \quad (27)$$

$$f_i^{(s+1,0)} = \sum_r \frac{\gamma_i^{(s,r,0)}}{R} \quad (28)$$

$$\mu_i^{(s+1)} = \frac{\sum_r \sum_t \gamma_i^{(s,r,t)} Y_t}{\sum_r \sum_t \gamma_i^{(s,r,t)}} \quad (29)$$

$$\sigma_i^{(s+1)} = \sqrt{\frac{\sum_r \sum_t \gamma_i^{(s,r,t)} \left(Y_t - \mu_i^{(s+1)}\right)^2}{\sum_r \sum_t \gamma_i^{(s,r,t)}}} \quad (30)$$

91
