# Supplementary material for: Estimating error rates for single molecule protein sequencing experiments
Source: PLoS Comput Biol. 2024 Jul 5;20(7):e1012258. doi: 10.1371/journal.pcbi.1012258 (PMC11253918; doi:10.1371/journal.pcbi.1012258)
Supplement: S2 Appendix — (PDF) [file pcbi.1012258.s002.pdf]

# S2 Appendix: Technical details of error type isolation

We now revisit Baum-Welch under the consideration of a factorization of the transition matrix, given as the product of $U$ matrix factors:

$$T^{(s)} = T^{(s,U)} T^{(s,U-1)} \dots T^{(s,1)} \tag{31}$$

Then we find that:

$$f^{(s,t)} = O^{(s,t)} T^{(s,U)} T^{(s,U-1)} \dots T^{(s,1)} f^{(s,t-1)} \tag{32}$$

We can think of these factors as transition probabilities between intermediate states. We define random variables of the form $X_{t,u}$ and letting$X_{t,0} = X_t$. We find that:

$$T_{ji}^{(s,u)} = p\left(X_{t,u} = j \middle| X_{t,u-1} = i, \theta^{(s)}\right) \tag{33}$$

It is useful to instead track intermediate cumulative values in this formulation. Let:

$$f^{(s,t,0)} = f^{(s,t)} \tag{34}$$

$$f^{(s,t,u+1)} = T^{(s,u+1)} f^{(s,t,u)} \tag{35}$$

Then it follows that:

$$f^{(s,t)} = O^{(s,t)} f^{(s,t-1,U)} \tag{36}$$

These values represent the probabilities of various states after sub-transitions, given as:

$$f_i^{(s,t,u)} = p\left(Y_{1:t} = y_{1:t}, X_{t,u} = i \middle| \theta^{(s)}\right) \tag{37}$$

Through a similar re-engineering of the backwards recursion we get:

$$b^{(s,t)} = {T^{(s,1)}}^{\top} {T^{(s,2)}}^{\top} \dots {T^{(s,U)}}^{\top} O^{(s,t+1)} b^{(s,t+1)} \tag{38}$$

$$b^{(s,t,U)} = O^{(s,t+1)} b^{(s,t+1)} \tag{39}$$

$$b^{(s,t,u)} = {T^{(s,u+1)}}^{\top} b^{(s,t,u+1)} \tag{40}$$

$$b^{(s,t)} = b^{(s,t,0)} \tag{41}$$

$$b_i^{(s,t,u)} = p\left(Y_{t+1:T} = y_{t+1:T} \middle| X_{t,u} = i, \theta^{(s)}\right) \tag{42}$$

We then need to track the probabilities of these intermediate states and transitions. We find them to be:

$$\gamma_i^{(s,t,u)} = \frac{f_i^{(s,t,u)} b_i^{(s,t,u)}}{f^{(s,t,u)^\top} b^{(s,t,u)}} \tag{43}$$

$$\xi_{ij}^{(s,t,u)} = \frac{f_i^{(s,t,u)} T_{ij}^{(s,u)} b_j^{(s,t,u+1)}}{f^{(s,t,u)^\top} T^{(s,u)} b^{(s,t,u+1)}} \tag{44}$$

Noting that $\xi^{(s,t,u)}$ is only defined when $u < U$, we then update our factored transition probabilities using the equation:

$$T_{ij}^{(s+1,u)} = \frac{\sum_t \xi_{ij}^{(s,t,u)}}{\sum_t \gamma_i^{(s,t,u)}} \tag{45}$$

The initial conditions and emission matrices can be computed as before, if we note that:

$$\gamma_i^{(s,t)} = \gamma_i^{(s,t,0)} \tag{46}$$

However, our initial conditions and emission probabilities have structure for fluorosequencing which will be helpful to define. In particular, our initial conditions are affected only by the missing fluorophore rate and the initial-blocking rate. We find it easiest to pretend we start with a perfectly labeled and non-blocked peptide, and then we apply a pre-transition which is different from the one used between emissions. Letting $\tau$ represent the pre-transition, we then use this pre-transition in the following manner:

$$f^{(s,0)} = \tau^{(s)} f^{(s,-1)} \tag{47}$$

$$b^{(s,-1)} = \tau^{(s)^\top} b^{(s,0)} \tag{48}$$

Letting $f_j^{(s,-1)}$ be 1 for the perfectly labeled and non-blocked state, and 0 everywhere else, while allowing the factorization $\tau^{(s)} = \tau^{(s,V)} \tau^{(s,V-1)} \dots \tau^{(s,1)}$ as was done with $T^{(s)}$ previously. The transition probabilities of $\tau^{(s)}$ can then be computed in manner just like that for $T^{(s)}$:

$$\tau_{ij}^{(s+1,u)} = \frac{\xi_{ij}^{(s,-1,u)}}{\gamma_i^{(s,-1,u)}} \tag{49}$$

We also consider the handling of $O^{(s,t)}$. In particular our output space is multidimensional, such that $Y_t \in \mathbb{R}^W$, so we let $Y_t = (Y_{t,1}, Y_{t,2}, \dots, Y_{t,W})$. If these components of the output are independent random variables, we can let:

$$O_{ii}^{(s,t,w)} = p\left(Y_{t,w} = y_{t,w} \middle| X_t = i, \theta^{(s)}\right) \tag{50}$$

$$O_{ii}^{(s,t)} = O_{ii}^{(s,t,1)} O_{ii}^{(s,t,2)} \dots O_{ii}^{(s,t,W)} \tag{51}$$

And we update our emission matrices as before. Assuming a normal distribution gives us:

$$\mu_i^{(s+1,w)} = \frac{\sum_t \gamma_i^{(s,t)} Y_{t,w}}{\sum_t \gamma_i^{(s,t)}} \tag{52}$$

$$\sigma_i^{(s+1,w)} = \sqrt{\frac{\sum_t \gamma_i^{(s,t)} \left(Y_{t,w} - \mu_i^{(s+1,w)}\right)^2}{\sum_t \gamma_i^{(s,t)}}} \tag{53}$$

$$O_{ii}^{(s+1,t,w)} = N\left(y_{t,w}; \mu_i^{(s+1,w)}, \sigma_i^{(s+1,w)}\right) \tag{54}$$

We can still generalize this to more sequences, using the generalized equations:

$$T_{ij}^{(s+1,u)} = \frac{\sum_r \sum_t \xi_{ij}^{(s,r,t,u)}}{\sum_r \sum_t \gamma_i^{(s,r,t,u)}} \tag{55}$$

$$\tau_{ij}^{(s+1,u)} = \frac{\sum_r \xi_{ij}^{(s,r,-1,u)}}{\sum_r \gamma_i^{(s,r,-1,u)}} \tag{56}$$

$$\mu_i^{(s+1,w)} = \frac{\sum_r \sum_t \gamma_i^{(s,r,t)} Y_{t,w}}{\sum_r \sum_t \gamma_i^{(s,r,t)}} \tag{57}$$

$$\sigma_i^{(s+1,w)} = \sqrt{\frac{\sum_r \sum_t \gamma_i^{(s,r,t)} \left(Y_{t,w} - \mu_i^{(s+1,w)}\right)^2}{\sum_r \sum_t \gamma_i^{(s,r,t)}}} \tag{58}$$

64
