# Supplementary material for: Estimating error rates for single molecule protein sequencing experiments
Source: PLoS Comput Biol. 2024 Jul 5;20(7):e1012258. doi: 10.1371/journal.pcbi.1012258 (PMC11253918; doi:10.1371/journal.pcbi.1012258)
Supplement: S3 Appendix — (PDF) [file pcbi.1012258.s003.pdf]

S3 Appendix: Technical details of maximum likelihood estimation

General formulas

Instead of getting transition probabilities, we may prefer to get a direct maximum likelihood estimate of a more fundamental parameter value that provides a more interpretable result. Furthermore, by involving a stricter structure in our formulation, parameterized by less parameters, our results should be less prone to error from overfitting the data. We will achieve this by means of a technique resembling what is done to estimate the parameterization of the continuous distributions for the emission matrix.

For every transition factor indexed by u, we introduce a parameter $p_{s+1,u}$, a function, $g_u : \mathbb{N} \to \mathbb{N}$, a function $h_u : \mathbb{N} \times \mathbb{N} \to \mathbb{N}$, and a function $\psi_u : \mathbb{R} \to \mathbb{R}^{N\times N}$. The form of these functions depends on the form of the random variable being modeled by the transition factor, and we will describe this in more detail below. For now we define:

$$p_{s+1,u} = \frac{\sum_r \sum_t \sum_i \sum_j \xi_{ij}^{(s,r,t,u)} h_u(i,j)}{\sum_r \sum_t \sum_i \gamma^{(s,r,t,u)} g_u(i)} \tag{59}$$

$$T^{(s+1,u)} = \psi_u\left(p_{s+1,u}\right) \tag{60}$$

Similarly for our initial conditions we let:

$$\hat{p}_{s+1,u} = \frac{\sum_r \sum_t \sum_i \sum_j \xi_{ij}^{(s,r,-1,u)} \hat{h}_u(i,j)}{\sum_r \sum_t \sum_i \gamma^{(s,r,-1,u)} \hat{g}_u(i)} \tag{61}$$

$$\tau^{(s+1,u)} = \hat{\psi}_u\left(\hat{p}_{s+1,u}\right) \tag{62}$$

Now we can define our factor specific functions.

Detachment rate estimation

We start with the detachment rate because it is the easiest to describe. It is defined by a Bernoulli random variable, which determines the rate at which a peptide enters a detached state instead of remaining unchanged.

We can only measure the detachment rate from states with remaining amino acids. We therefore set $g_u(i)$ to 1 for those states, and 0 for the remaining state. To pick up the transition rate into these states, we set $h_u(i,j)$ to 1 if i has remaining amino acids *and* j indicates the detached state. We set it to 0 for all other combinations of i and j, which should never occur as they should have been forbidden in the definition of the transition factor in the previous iteration of Baum-Welch, $T^{(s,u)}$.

This is a weighted maximum likelihood estimate of a Bernoulli random variable. We use the resulting value of $p_{s+1,u}$ to define the corresponding transition factor. ψ_u then defines $T_{ij}^{(s+1,u)}$ to be $p_{s+1,u}$ when i has remaining amino acids *and* j indicates the detached state, and to be $1 - p_{s+1,u}$ when $i = j$ and they are not in the detached state. When both are in the detached state, $T_{ij}^{(s+1,u)}$ is 1, and for all other combinations of i and j, we set $T_{ij}^{(s+1,u)}$ to 0.

N-terminal blocking rate estimation

N-terminal blocking behavior is also defined by Bernoulli random variables. There are two of these rates, and we start by defining our factor-specific functions for the cyclic blocking behavior, which defines the rate at which a peptide moves into a blocked state instead of remaining unchanged.

We can only measure this rate from the unblocked states. Let $g_u(i) = 1$ in those states, and 0 for all other states. $h_u(i,j) = 1$ if i represents an unblocked state and j indicates the specifically corresponding blocked state. $h_u(i,j) = 0$ for all other combinations of i and j, though these should never occur under a correct implementation of this algorithm.

This again is a weighted maximum likelihood estimate of a Bernoulli random variable, and we can describe the action of ψ_u. $T_{ij}^{(s+1,u)}$ should be $p_{s+1,u}$ when i is an unblocked state and j is the corresponding blocked state, while it should be $1 - p_{s+1,u}$ when $i = j$ and they are in an unblocked state. When both are in the same unblocked state, $T_{ij}^{(s+1,u)}$ is 1, and for all other combinations of i and j, we set $T_{ij}^{(s+1,u)}$ to 0.

For the matrix factor representing the initial N-terminal blocking, we analyze the data in exactly the same way, but just use a different parameter and matrix to track our results, in order to allow the initial blocking rate to be different from the cyclic one.

Dye destruction rate estimation

An assumption of equal exposure to both chemical failure and photobleaching of the fluorophores means we should treat this as a binomial random variable. As with N-terminal blocking rates, we have one rate that identifies the behavior before sequencing starts (the missing fluorophore rate), and another for destruction during sequencing (the dye loss rate). These will again be mostly equivalent in their analysis, though tracked with different variables to enforce a separation. We show here the analysis for the dye loss rate. We also wish to emphasize that we track these separately, and with separate functions g_u, h_u, and ψ_u, for each color of fluorophore.

We note that states with more fluorophores of the color being analyzed provide more evidence of the rate of fluorophore loss. With this in mind, $g_u(i)$ is set to the number of fluorophores of the color of interest in state i. We then let $h_u(i,j) = g_u(i) - g_u(j)$ if $j \leq i$, and otherwise set it to 0 (for transitions which should never occur).

To construct $T^{(s+1,u)}$, we let $T_{ij}^{(s+1,u)} = B\left(h_u(i,j); g_u(i), p_{s+1,u}\right)$ if $j \leq i$, where B represents the parameterized probability mass function of the binomial distribution. This expands to:

$$T_{ij}^{(s+1,u)} = \binom{g_u(i)}{g_u(j)} \left(p_{s+1,u}\right)^{h_u(i,j)} \left(1 - p_{s+1,u}\right)^{g_u(j)} \quad (63)$$

When $j \leq i$. When $j > i$, we let $T_{ij}^{(s+1,u)} = 0$.

There is a key difference in the analysis of the missing fluorophore, or dud-dye, rate. A bias is introduced because peptides with all fluorophores missing in every color will not be visible and are therefore absent from the data. A correction for this bias is discussed later in this text.

Edman failure rate estimation

Our factored transitions which manage Edman degradation are defined by a Bernoulli random variable, much like the factors managing the detachment rate or the two forms of the N-terminal blocking rates. While there is an additional complication from the probabilistic loss of a fluorophore in the case of a successful Edman degradation, we can safely ignore this until we need to construct $T^{(s+1,u)}$. When determining $p_{s+1,u}$, we need only concern ourselves with whether Edman degradation failed.

We need to omit states with a blocked N-terminus. For all states with a blocked N-terminus, $g_u(i) = 0$. For states with an unblocked N-terminus, $g_u(i) = 1$. We let $h_u(i,j) = 0$ if either or both of i or j represent a state with a blocked N-terminus. We also set it to 0 for all invalid combinations of i and j that should never occur; this relationship is complicated and peptide dependent. Combinations of i and j are valid when $i = j$, or when removing the N-terminal amino acid from state i can result in state j. In the second case, state j represents either the same combination of fluorophore counts of different

colors but with one less amino acid, *or* it represents that combination less one amino acid *and* minus one fluorophore, *of the specific color* of fluorophore which may or may not be present on the amino acid being removed. This is invalid when the N-terminal amino acid cannot be labeled and in that case should be zero.

In any case, when $i = j$, we let $h_u(i,j) = 0$, as this is an Edman success. For all other *valid* combinations of i and j, we let $h_u(i,j) = 1$. This will give a weighted maximum likelihood estimate of the probability of an Edman failure event.

To define $T^{(s+1,u)}$, we let $T_{ij}^{(s+1,u)} = 1 - p_{s+1,u}$ when $i = j$. For $i \neq j$ (assuming a valid transition), if state i does not have a possibility of a fluorophore on its N-terminal amino acid, then $T_{ij}^{(s+1,u)} = p_{s+1,u}$. If a fluorophore is possible on its N-terminal amino acid, the computation is a bit more involved.

In [16], we derived a formula for the probability of fluorophore removal with Edman degradation, which we reiterate here under a slightly different symbolic representation. Let $\lambda(i)$ represent the number of labelable amino acids which can take a fluorophore of the same color that the N-terminal amino acid may have when in state i. Let $G(i)$ represent the number of fluorophores of the same color the N-terminal amino acid may have, when in state i. We note that this second function is similar in form to the variant of $g_u(i)$ described under "Dye destruction rate estimation." Now, in the case where $i \neq j$ (for a valid transition) and state i *does* have a possibility of an N-terminal amino acid, and j, in relation to i, represents an amino acid removal, we let:

$$T_{ij}^{(s+1,u)} = \frac{G(i)}{\lambda(i)} p_{s+1,u} \qquad (64)$$

Then, for the case where $i \neq j$ (for a valid transition) and state i has a possibility of an N-terminal amino acid, and j, in relation to i, represents no amino acid removal, we then let:

$$T_{ij}^{(s+1,u)} = \left(1 - \frac{G(i)}{\lambda(i)}\right) p_{s+1,u} \tag{65}$$

Finally, for invalid transitions, we let $T_{ij}^{(s+1,u)} = 0$.

Additional technical discussion of weighted parameter estimation

We note that, with the exception of Edman degradation, all of the functions $g_u(i)$ and $h_u(i,j)$ are trivial to compute when the cumulative forward and backward probability results are indexed by a higher-order tensor. Then, these functions become a simple extraction of an index of that higher-order tensor.
