# Supplementary material for: Estimating error rates for single molecule protein sequencing experiments
Source: PLoS Comput Biol. 2024 Jul 5;20(7):e1012258. doi: 10.1371/journal.pcbi.1012258 (PMC11253918; doi:10.1371/journal.pcbi.1012258)
Supplement: S4 Appendix — (PDF) [file pcbi.1012258.s004.pdf]

S4 Appendix: Technical details of bias correction

When a peptide is missing all its fluorophores it is not visible and does not get sequenced. Correcting for this issue involves considerable additional complexity. We start by noting that this introduces a statistical dependency between missing fluorophore rates of different colors of fluorophores. This is because a count of zero fluorophores of one color only eliminates the peptide from the dataset if there are also zero fluorophores of every other color. We find it clearer to first define $\tau^{(s+1,u)}$. As in the case for the dye loss rate, let $g_u(i)$ represent the number of fluorophores of the color of interest in state i, and let $h_u(i,j) = g_u(i) - g_u(j)$ if $j \leq i$, and otherwise set it to zero.

Now however, instead of defining $\tau^{(s+1,u)}$ with a parameterized binomial distribution as we did before for $T^{(s+1,u)}$, we use a modified binomial distribution where the entries represent the probability of the transition *conditioned on* the probability of the peptide being observable. Letting $\hat{C}$ represent the set of all pre-sub-transition indices corresponding to missing fluorophore rates of different fluorophore colors, this equation is given by:

$$\tau_{ij}^{(s+1,u)} = \frac{B\big(h_u(i,j); g_u(i), \hat{p}_{s+1,u}\big)}{1 - \prod_{\hat{u}\in\hat{C}} B\big(0; g_{\hat{u}}(i), \hat{p}_{s+1,\hat{u}}\big)} \qquad (66)$$

We of course require, as before, $j \leq i$, and otherwise set $\tau_{ij}^{(s+1,u)}$ to zero. Additionally, we note that $\tau_{ij}^{(s+1,u)}$ should be zero for the state i such that $g_u(i) = 0$ when $\hat{u} \in \hat{C}$ (the state with no fluorophores of any color).

With other forms of error, we could point to existing and well understood formulas for their maximum likelihood estimates, as they can be viewed either as binomial or Bernoulli random variables. That will not work in this case, and we must explicitly derive

this result. We consider the colors of fluorophore together, due to their statistical dependencies. Then we seek to maximize the likelihood given by:

$$L = \prod_i \prod_j \left(\frac{\prod_{\hat{u}\in\hat{C}} B\left(h_{\hat{u}}(i,j); g_{\hat{u}}(i), \hat{p}_{s+1,\hat{u}}\right)}{1 - \prod_{\hat{u}\in\hat{C}} B\left(0; g_{\hat{u}}(i), \hat{p}_{s+1,\hat{u}}\right)} \right)^{\Xi_{ij}^{(s,r,-1,\hat{C})}} \tag{67}$$

Assuming i and j vary over only their valid ranges, and where we let $\Xi_{ij}^{(s,r,-1,\hat{C})}$ represent the probability of a transition from state i to state j given $Y_{1:T} = y_{1:T}$ and $\theta^{(s)}$ (for iteration s, sequence r, time step t) when considering only the subset of sub-transitions contained in U, the set of all missing fluorophore related sub-sequences. In mathematical notation:

$$\Xi_{ij}^{(s,r,t,U)} = \prod_{u\in U} \xi^{(s,r,t,u)} \tag{68}$$

We now note a useful simplification to our formula. We previously defined our initial conditions for $f^{(s,-1)}$ so that it would be one for the perfectly labeled and non-blocked state, and zero everywhere else. The only pre-sub-transition other than the missing fluorophore rate is the transition for initial N-blocking. The status of the N-terminus is irrelevant to the missing fluorophore rate, thus we consider the blocked and unblocked states together. We also ignore all states with missing fluorophores as irrelevant. We name the remaining state I. Then we can reduce our likelihood equation to:

$$L = \prod_j \left(\frac{\prod_{\hat{u}\in\hat{C}} B\left(h_{\hat{u}}(I,j); g_{\hat{u}}(I), \hat{p}_{s+1,\hat{u}}\right)}{1 - \prod_{\hat{u}\in\hat{C}} B\left(0; g_{\hat{u}}(I), \hat{p}_{s+1,\hat{u}}\right)} \right)^{\Xi_{Ij}^{(s,r,-1,\hat{C})}} \tag{69}$$

Expanding our equation for the likelihood we get:

$$L = \prod_j \left(\frac{\prod_{\hat{u}\in\hat{C}} \binom{g_{\hat{u}}(I)}{g_{\hat{u}}(j)} \left(\hat{p}_{s+1,\hat{u}}\right)^{h_{\hat{u}}(I,j)} \left(1 - \hat{p}_{s+1,\hat{u}}\right)^{g_{\hat{u}}(j)}}{1 - \prod_{\hat{u}\in\hat{C}} \left(\hat{p}_{s+1,\hat{u}}\right)^{g_{\hat{u}}(I)}} \right)^{\Xi_{Ij}^{(s,r,-1,\hat{C})}} \tag{70}$$

We now take the logarithm, which makes the equation easier to work with while preserving order:

$$\log(L) = \sum_j \Xi_{Ij}^{(s,r,-1,\hat{C})} \left(\sum_{\hat{u}\in\hat{C}} \left(\log\binom{g_{\hat{u}}(I)}{g_{\hat{u}}(j)} + h_{\hat{u}}(I,j)\log\left(\hat{p}_{s+1,\hat{u}}\right) + g_{\hat{u}}(j)\log\left(1-\hat{p}_{s+1,\hat{u}}\right)\right) - \log\left(1-\prod_{\hat{u}\in\hat{C}}\left(\hat{p}_{s+1,\hat{u}}\right)^{g_{\hat{u}}(I)}\right)\right) \tag{71}$$

Noting that this equation is symmetric with respect to $\hat{u}$, we need only solve for one result to maximize for every $\hat{p}_{s+1,\hat{u}}$. We then take the derivative with respect to a choice of $\hat{p}_{s+1,\hat{u}}$ and set it to zero to search for extrema.

$$0 = \sum_j \Xi_{Ij}^{(s,r,-1,\hat{C})} \left(\frac{h_u(I,j)}{\hat{p}_{s+1,u}} - \frac{g_u(j)}{1-\hat{p}_{s+1,u}} + \frac{g_u(I)\prod_{\hat{u}\in\hat{C}}\left(\hat{p}_{s+1,\hat{u}}\right)^{g_{\hat{u}}(I)}}{\hat{p}_{s+1,u}\left(1-\prod_{\hat{u}\in\hat{C}}\left(\hat{p}_{s+1,\hat{u}}\right)^{g_{\hat{u}}(I)}\right)} \right) \tag{72}$$

This reduces to:

$$0 = \sum_j \Xi_{Ij}^{(s,r,-1,\hat{C})} \left(h_u(I,j) - \left(\hat{p}_{s+1,u}\right)g_u(I) - \left(1-\hat{p}_{s+1,u}\right)g_u(I)\left(1-\left(1-\prod_{\hat{u}\in\hat{C}}\left(\hat{p}_{s+1,\hat{u}}\right)^{g_{\hat{u}}(I)}\right)\right)\right) \tag{73}$$

And reduces again to:

$$0 = \sum_j \Xi_{Ij}^{(s,r,-1,\hat{C})} \left(g_u(j) - \frac{\left(1-\hat{p}_{s+1,u}\right)g_u(I)}{1-\prod_{\hat{u}\in\hat{C}}\left(\hat{p}_{s+1,\hat{u}}\right)^{g_{\hat{u}}(I)}} \right) \tag{74}$$

If we rearrange, we find:

$$\frac{1-\hat{p}_{s+1,u}}{1-\prod_{\hat{u}\in\hat{C}}\left(\hat{p}_{s+1,\hat{u}}\right)^{g_{\hat{u}}(I)}} = \frac{\sum_j \Xi_{Ij}^{(s,r,-1,\hat{C})} g_u(j)}{g_u(I)} \tag{75}$$

This is a multivariate generalization of the solution to a geometric sequence and is therefore a generalization of the associated inverse problem. This also constitutes root-finding of a polynomial of arbitrary order, and thus is unlikely to have a closed form solution. If we assume large $g_{\hat{u}}(I)$ or small $\hat{p}_{s+1,\hat{u}}$ for at least one color of fluorophore, then this can be approximated by:

$$\hat{p}_{s+1,u} \approx \frac{\sum_j \Xi_{Ij}^{(s,r,-1,\hat{C})} h_u(I,j)}{g_u(I)} \tag{76}$$

This is the maximum likelihood estimate for an ordinary binomial random variable. We use this to demonstrate that the extremum we've found is a maximum, in place of applying the second derivative test as would ordinarily be done.

We need a way to approximately solve this when $g_{\hat{u}}(I)$ is small or $\hat{p}_{s+1,\hat{u}}$ is large. We suggest an iterative method, where we plug the left-hand side result into the right-hand side on each iteration. We iterate over z, writing our new equation as:

$$\hat{p}_{s+1,u}^{(z+1)} = 1 - \frac{\left(1 - \prod_{\hat{u}\in\hat{C}} \left(\hat{p}_{s+1,\hat{u}}^{(z)}\right)^{g_{\hat{u}}(I)}\right) \sum_j \Xi_{Ij}^{(s,r,-1,\hat{C})}\, g_u(j)}{g_u(I)} \tag{77}$$

We need to prove that this iteration converges. The multivariate nature of this problem requires us to consider this in a multivariate vector space of dimension $\left|\hat{C}\right|$. We also set some requirements. Firstly, this equation is clearly nonsense unless:

$$\sum_{u\in\hat{C}} g_u(I) \geq 2 \tag{78}$$

We also require a constraint on the solution:

$$0 < \hat{p}_{s+1,\hat{u}} < 1 \tag{79}$$

For convenience, we now introduce the following variables:

$$P_{s+1}^{(z)} = \prod_{\hat{u}\in\hat{C}} \left(\hat{p}_{s+1,u}^{(z)}\right)^{g_{\hat{u}}(I)} \tag{80}$$

$$P_{s+1} = \prod_{\hat{u}\in\hat{C}} \left(\hat{p}_{s+1,\hat{u}}\right)^{g_{\hat{u}}(I)} \tag{81}$$

$$0 < S_{s+1,u} = \frac{1 - \hat{p}_{s+1,u}}{1 - P_{s+1}} = \frac{\sum_j \Xi_{Ij}^{(s,r,-1,\hat{C})}\, g_u(j)}{g_u(I)} < 1 \tag{82}$$

This gives us the equation:

$$\hat{p}_{s+1,u}^{(z+1)} = 1 - \left(1 - P_{s+1}^{(z)}\right) S_{s+1,u} \tag{83}$$

Equivalently:

$$\hat{p}_{s+1,u}^{(z+1)} = 1 - S_{s+1,u} + S_{s+1,u} P_{s+1}^{(z)} \tag{84}$$

If we have a vector of $\hat{p}_{s+1,u}^{(z)}$ such that $\hat{p}_{s+1,u}^{(z)} = P_{s+1}$, then clearly from this equation we will get the correct result in the next iteration, and $\hat{p}_{s+1,u}^{(z+1)} = \hat{p}_{s+1,\hat{u}}$. More generally, due to the linear relationship of these terms, and noting that $0 < S_{s+1,u} < 1$, if $P_{s+1}^{(z)} = P_{s+1} + \epsilon$ for $|\epsilon| \ll 1$, then there exists $|\delta| < |\epsilon|$ such that $\hat{p}_{s+1,u}^{(z+1)} = \hat{p}_{s+1,\hat{u}} + \delta$. Therefore, to prove convergence of every $\hat{p}_{s+1,u}^{(z)}$ to $\hat{p}_{s+1,u}$ for all $u \in \hat{C}$ it is sufficient to prove convergence of $P_{s+1}^{(z)}$ to P_{s+1}.

There is a recurrence relation of $P_{s+1}^{(z+1)}$ in terms of $P_{s+1}^{(z)}$, which is:

$$P_{s+1}^{(z+1)} = \prod_{u \in \hat{C}} \left(1 - S_{s+1,u} + S_{s+1,u} P_{s+1}^{(z)}\right)^{g_u(I)} \tag{85}$$

The right-hand side is a polynomial expression of order $\sum_{u\in\hat{C}} g_u(I)$ with all positive coefficients. It follows that its derivatives of every order up to and including $\sum_{u\in\hat{C}} g_u(I)$ are strictly positive. Our previous requirement that $\sum_{u\in\hat{C}} g_u(I) \geq 2$ implies that the polynomial is strictly positive and has strictly positive first and second derivatives for $0 < P_{s+1}^{(z)} < 1$. The strictly positive second derivative in this range guarantees that no more than two fixed points are possible in the given range. There is by definition a fixed-point solution $0 < P_{s+1} < 1$, which is the solution we want our iteration to converge towards. There is also another fixed point at 1.

Noting that for $P_{s+1}^{(z)} = 0$ we get $P_{s+1}^{(z+1)} > 0$, it follows that:

$$P_{s+1}^{(z+1)} > P_{s+1}^{(z)} \text{ for } 0 < P_{s+1}^{(z)} < P_{s+1} \tag{86}$$

A double root at P_{s+1} is incompatible with the fixed point at 1. Therefore:

$$P_{s+1}^{(z+1)} < P_{s+1}^{(z)} \text{ for } P_{s+1} < P_{s+1}^{(z)} < 1 \tag{87}$$

Using the strictly positive first derivative, we also find that both:

$$P_{s+1}^{(z+1)} < P_{s+1} \text{ for } 0 < P_{s+1}^{(z)} < P_{s+1} \tag{88}$$

$$P_{s+1}^{(z+1)} > P_{s+1} \text{ for } P_{s+1} < P_{s+1}^{(z)} < 1 \tag{89}$$

These four results can be combined to prove the relation:

$$\left|P_{s+1}^{(z+1)} - P_{s+1}\right| < \left|P_{s+1}^{(z)} - P_{s+1}\right| \tag{90}$$

Therefore, given any $0 < P_{s+1}^{(0)} < 1$ our iteration eventually converges towards the desired result.

We then fold this iteration into the outer iteration used to improve all estimates in the Baum-Welch algorithm, which gives the equation:

$$\hat{p}_{s+1,u} = 1 - \frac{\left(1 - \prod_{\hat{u}\in\hat{C}}\left(\hat{p}_{s,\hat{u}}\right)^{g_{\hat{u}}(I)}\right)\sum_j \Xi_{Ij}^{(s,r,-1,\hat{C})}\, g_u(j)}{g_u(I)} \tag{91}$$

This is equivalent to imputing missing data using the parameter estimates in the previous iteration, as described in the nontechnical summary of this bias correction. We also note that it is tempting to apply a root finding method from an open-source library, which would likely converge faster in practice. However, our method allows us to accommodate cross-parameter effects by default, which is a desirable property. There is of course a valid concern that cross-parameter effects could in some way invalidate this proof, but we have found in practice that this does not appear to be the case.
