# Supplementary material for: Estimating error rates for single molecule protein sequencing experiments
Source: PLoS Comput Biol. 2024 Jul 5;20(7):e1012258. doi: 10.1371/journal.pcbi.1012258 (PMC11253918; doi:10.1371/journal.pcbi.1012258)
Supplement: Attachment — Submitted filename: whatprot-fit, responses to reviewer comments-1.pdf [file pcbi.1012258.s005.pdf]

Responses to reviewer comments for paper: Estimating error rates for single molecule protein sequencing experiments

Our responses are written inline in blue text.

Reviewer #1: The authors present a methodology to analyze SMPS read data and estimate the error rate of reads. To estimate the error rate, from different sources, they adapted the Baum-Welch algorithm. The method was evaluated on both simulated and experimental data.

We thank the reviewer for their time in reviewing this manuscript, and their insightful comments which will lead to an improved version of the paper.

While I think the method is going to be very valuable for the field, the current manuscript mainly lacks in showing its application potential. Could the authors address the points outlined below?

1. As the authors indicate, there are discrepancies between the simulated and experimental data and estimates of the error rates. In the worst case, this suggests that the simulated data is incorrect and cannot be used to evaluate methods presented here. Is there any procedure the authors went through to validate the simulated data? In the discussion, currently, the question of the cause of this discrepancy is kept open. I think the authors should further investigate this or leave the simulation part out of the manuscript. As it is not clear to me whether this data is actually valid and can be used.

Both of the fitting procedures presented here require an explicitly-defined model. The model we described is based upon a combination of (1) intuition about the chemistry being applied during sequencing developed over several years of experimental runs, and (2) visual analysis of histograms of data from experimental runs, particularly discrepancies of such histograms with histograms fit by our fitting procedures. While we attempt to identify all forms of error, and are confident we have identified the largest error sources, it seems inevitable that some forms of error have escaped us. We expect that they would be incorporated in the future in a manner similar to what we've described in this paper. We have now clarified this in the text on lines 118-126.

Despite potential missing parameters, fitting on simulated data serves to validate that, if the model were completely correct, the fit will generally be accurate. While it does not guarantee the quality of the fit on experimental data, it raises our confidence. Consider the alternative; if the fit did not work correctly on simulated data that we know to be of the same underlying model, could a fit on experimental data be trusted? We have addressed this in the text on lines 484-488.

2. To me it seems that the authors mainly focused on the stability/reproducibility of their error estimates. To me it is unclear if the authors validate these numbers to be correct in their

manuscript. Also, I do not see a clear application for the current state of the model, could the authors go more in-depth on how these error-estimates should be used? It would also be great if the authors show an application of the model.

Because fluorosequencing is a new technology, methods to determine the true values of these parameters in experimental data are not yet well developed. In previous publications we were able to isolate the dye loss rate experimentally by sequencing N-terminally blocked peptides, but the Edman failure rate could not be isolated because we do not know how to bring the dye loss rate to zero, however much we would like to; in previous publications we simply ignored this confounding factor. Similar issues exist for most of the other parameters. This paper, if published, will be the first to demonstrate determination of error rates using statistical techniques that can account for interactions between forms of error. We are unable to validate these error rates against true values because there are no pre-existing methods to determine them. This was, for us, a motivation to have more than one fitter, as agreement between the fitters improves our confidence in the estimates. We readily admit that such a technique is not foolproof. In the new revision we discuss this in the text on lines 329-336.

As to how these error estimates should be used: there are two applications.

(1) error-estimates help inform organic chemists working on the fluorosequencing technology in how various changes to the sequencing chemistry effect sequencing behavior (see for example **Figure 14**). In particular, these estimates should in theory be the same for different peptides. Without such a technique, it is possible to compare numbers of incorrect sequences for a peptide or even numbers of which incorrect sequences we get, and we have done this in the past, but such an error analysis focuses on secondary effects rather than the fundamental chemical processes, and fails to generalize to different peptides.

(2) better error-estimates improve classification performance. The ultimate goal of fluorosequencing is to sequence peptides, thus determining the protein concentrations in a biological sample. Applications would be akin to those of tandem mass spectrometry, though our emphasis is on smaller sample sizes with single-molecule sensitivity. Because collecting large training sets for, e.g., each of the nearly one-million peptides in a trypsinized human proteome, would be prohibitively expensive, our machine learning classifiers either use the parameterization directly, or train on simulated datasets based on the same parameterized model. With better error-estimates we expect to improve the performance of such classifiers.

Both points are addressed in the text on lines 127-137.

3. The authors mention that their implementation of Baum-Welch is more robust to overfitting, but to me this is not apparent from the manuscript.

We see that as currently written, this statement was unclear and misleading. We meant only to claim that our modified Baum-Welch implementation should be less prone to overfitting than the classical Baum-Welch algorithm for this particular problem. Consider Figure 3 in which we can

see 42 states for a relatively small peptide with only 3 fluorophores. The classical Baum-Welch algorithm would fit a transition probability from each state to each state, for a total of 42*42=1764 parameters. Larger peptides could have orders of magnitude more states. By modifying the Baum-Welch algorithm as described in the text, we reduce this to just 6 parameters. While the parameters we have chosen may not be fully accurate or comprehensive, such a dramatically reduced parameter space is known to significantly reduce risk and severity of overfitting. We have removed this statement from the abstract (lines 32-33), and improved our statement on line 184 to state this more effectively.

Reviewer #2: The authors provided an advanced method to estimate the parameters or error rates for single-molecule protein sequencing. This method extended the Baum-Welch algorithm to the previous algorithm Whatprot model which is based on the HMM model to perform peptide classification on florosequencing data. The Baum-Welch algorithm is adapted to make use of the forward-backward algorithm to maximize the likelihood by finding the unknown parameters in the HMM model. The authors demonstrated the high accuracy of parameter estimation on simulated by adopting the Baum-Welch algorithm in the HMM model. Meanwhile, the authors provided a second option using DIRECT and Powell's method to reduce the RMSE which also has been proven on simulation and real datasets.

We thank the reviewer for taking the time to review this manuscript, and appreciate the positive response.

The paper showed a clear idea about the method and solid results to support the application. The authors gave comprehensive mathematical model explanation and detailed proof. Overall, it's well-prepared to be accepted. Here are only some minor suggestions.

1) The main figure 1 depicts the essential steps of single-molecule protein sequencing and labels the potential error rates of the steps, which are the parameters that need to be estimated by the HMM model. The caption gives a detailed technical explanation of Figure 1 from the chemistry and sequencing aspects. However, there are lack of demonstration to build the mathematical model with real steps in fluorosequencing. For example, where/which steps are the hidden states generated from? What does transition_probability/ emission_probability represent in those steps? What are the observations? The mathematical notations representations are essential to help readers to understand how to build the model.

Please see our response to #2

2) The same problem existed in Figure 2, especially, since there is no clear explanation/notations to demonstrate how the hidden Markov model applies for fluorosequencing. Could you please give a simple example to clearly show how the HMM model matches the SMPS?

In re-reading our work, we agree that we failed to effectively communicate the connection between the physical fluorosequencing process and the hidden Markov model we present. We

have now added a clarifying figure (Figure 2 in the new draft) as well as an explanation in the main text (various edits lines 147 through 157) that should address this shortcoming. We hope that this adequately addresses your concerns.

3) In the paper, there are multiple times mentioned “as in Chapter 2”, for example, line 158, line 171, line 1032… Is there any missed literature that needs to be cited?

Corrected. This should have referred to reference [16] Smith MB *et al.* Amino Acid sequence assignment from single molecule peptide sequencing data using a two-stage classifier.

4) In line 170, “illustration from Figure 2.4 to include N-terminal blocking ”, I could not find Figure 2.4, please correct the Figure citation.

Corrected.
